# The Methylation Analysis of the *Glucose-Dependent Insulinotropic Polypeptide Receptor (GIPR)* Locus in GH-Secreting Pituitary Adenomas

**DOI:** 10.3390/ijms24119264

**Published:** 2023-05-25

**Authors:** Mattia Dalle Nogare, Sarah D’Annunzio, Giovanni Vazza, Daniela Regazzo, Luna Picello, Luca Denaro, Giacomo Voltan, Carla Scaroni, Filippo Ceccato, Gianluca Occhi

**Affiliations:** 1 Department of Biology, University of Padova, 35128 Padova, Italy; mattia.dallenogare@phd.unipd.it (M.D.N.);; 2 Department of Cellular, Computational and Integrative Biology (CIBIO), University of Trento, 38123 Trento, Italy; 3 Endocrinology Unit, Department of Medicine, Padova University Hospital, 35121 Padova, Italy; 4 Academic Neurosurgery, Department of Neurosciences, Padova University Hospital, 35121 Padova, Italy

**Keywords:** glucose-dependent insulinotropic polypeptide receptor (GIPR), GH-secreting pituitary adenomas, CpG methylation, 5-aza-2′-deoxycytidine, acromegaly

## Abstract

The glucose-dependent insulinotropic polypeptide receptor (GIPR) is aberrantly expressed in about one-third of GH-secreting pituitary adenomas (GH-PAs) and has been associated with a paradoxical increase of GH after a glucose load. The reason for such an overexpression has not yet been clarified. In this work, we aimed to evaluate whether locus-specific changes in DNA methylation patterns could contribute to this phenomenon. By cloning bisulfite-sequencing PCR, we compared the methylation pattern of the GIPR locus in GIPR-positive (GIPR^+^) and GIPR-negative (GIPR^−^) GH-PAs. Then, to assess the correlation between *Gipr* expression and locus methylation, we induced global DNA methylation changes by treating the lactosomatotroph GH3 cells with 5-aza-2′-deoxycytidine. Differences in methylation levels were observed between GIPR^+^ and GIPR^−^ GH-PAs, both within the promoter (31.9% vs. 68.2%, *p* < 0.05) and at two gene body regions (GB_1 20.7% vs. 9.1%; GB_2 51.2% vs. 65.8%, *p* < 0.05). GH3 cells treated with 5-aza-2′-deoxycytidine showed a ~75% reduction in *Gipr* steady-state level, possibly associated with the observed decrease in CpGs methylation. These results indicate that epigenetic regulation affects GIPR expression in GH-PAs, even though this possibly represents only a part of a much more complex regulatory mechanism.

## 1. Introduction

The glucose-dependent insulinotropic polypeptide receptor (GIPR) is a class B G-protein coupled receptor [1] with a wide range of functions in different tissues and physiological processes. These include the potentiation of glucose-dependent insulin secretion in pancreatic β-cells, lipid uptake and lipogenesis in fatty tissue, neurogenesis, and neuronal survival in the brain [2,3,4].

In addition to its association with some metabolic disorders [5,6], the GIP/GIPR axis has inspired increasing interest because of its inappropriate expression and activation in certain human endocrine tumors [7,8], where it may have both diagnostic and prognostic significance [7]. In food-dependent Cushing’s syndrome (FDCS), the ectopic expression of GIPR has been associated with the inversed rhythm of cortisol secretion [9], while in neuroendocrine tumors (NET), the incidence/density of GIPR may correlate with increasing proliferative rates [8,10] and the development of metastasis [11]. In acromegaly, GIPR overexpression in GH-secreting pituitary adenomas (GH-PAs) correlates both with clinical and molecular features. In addition to the lack of the *gsp* oncogene, GIPR positive (GIPR^+^) somatotropinomas are characterized by a distinctive transcriptional profile and a higher frequency of genomic rearrangements and hypermethylation [12,13,14]. From a clinical point of view, GIPR mediates, in most cases, the paradoxical increase of GH after glucose load, which in turn is associated with a milder tumoral phenotype. GH-PAs that develop in a ‘paradoxical background’ are, indeed, smaller, less invasive, and show a better response to medical therapy [15,16,17,18].

Recent evidence suggests that the aberrant expression of GIPR in a subset of endocrine tumors may be a secondary event rather than a driver event in tumorigenesis [8], and a consequence of epigenetic rather than genetic abnormalities [11,19]. Recent studies have demonstrated that germline-inactivating mutations in the *KDM1A* demethylase encoding gene contribute to the abnormal expression of GIPR in adrenal lesions of patients with FDCS [19,20]. Aberrant methylation of the *GIPR* locus is instead associated with upregulation of GIPR in NETs of the small intestine [11]. In the context of GH-PAs, there is no evidence of recurrent genetic events that sustain GIPR overexpression [12,13,21]. However, a role for altered methylation in mediating this phenomenon has been proposed [12].

Considering these premises, in the current study we aimed to investigate whether altered epigenetic control of the GIPR locus acts as a driving force for its overexpression in GH-PAs. Elucidating the molecular causes underlying this phenomenon may aid to advance our understanding of GH-PAs pathogenesis and provide insights into the heterogeneity that characterizes this tumor type.

## 2. Results

### 2.1. Methylation of the Promoter and the Gene Body Correlate with GIPR Expression in GH-PAs

**Figure 1:**
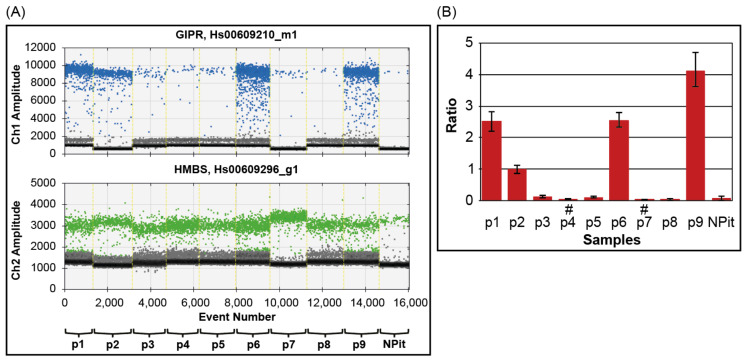
The relative expression of *GIPR* in GH-PAs was evaluated via ddPCR. (**A**) Steady-state level of *GIPR* (upper panel) and *HMBS* (lower panel) in GH-PAs (p1–p9) and a normal pituitary gland (NPit). One-dimensional-dot plot display of mono-color droplet fluorescence intensity. Colored and black dots indicate each droplet positive (blue for GIPR, green for HMBS) or negative for PCR assay, respectively. For each sample, droplets are depicted according to the event (number of droplets read during reading; x-axis) and its FAM (Blue) or VIC (green) fluorescence intensity (Ch1/Ch2 Amplitude; y-axis). The setting of the threshold for droplet positivity was performed manually. (**B**) The normalized ratio of GIPR to HMBS in all samples. The error bars associated with each point represent Poisson’s 95% confidence intervals. #, *gsp*^+^.

We evaluated the steady-state level of *GIPR* in nine selected somatotropinomas and one normal pituitary gland using droplet digital PCR (ddPCR). To this end, TaqMan GIPR and HMBS (hydroxymethylbilane synthase) hydrolysis probes were used, the latter selected as the most stable housekeeping gene in pituitary adenomas [22]. With a cutoff based on the observed range of GIPR expression for normal pituitary glands [21], the analyzed GH-PAs were divided into two distinct subgroups (see Figure 1). The first group, referred to as GIPR^−^, comprised p3, p4, p5, p7, and p8, and expressed *GIPR* (0.027 to 0.124, mean 0.061 ± 0.047) at levels comparable to the normal pituitary gland (0.07). The second group (GIPR^+^), with p1, p2, p6, and p9, expressed *GIPR* at significantly higher levels (from 0.98 to 4.1, mean 2.535 ± 1.274).

**Figure 2:**
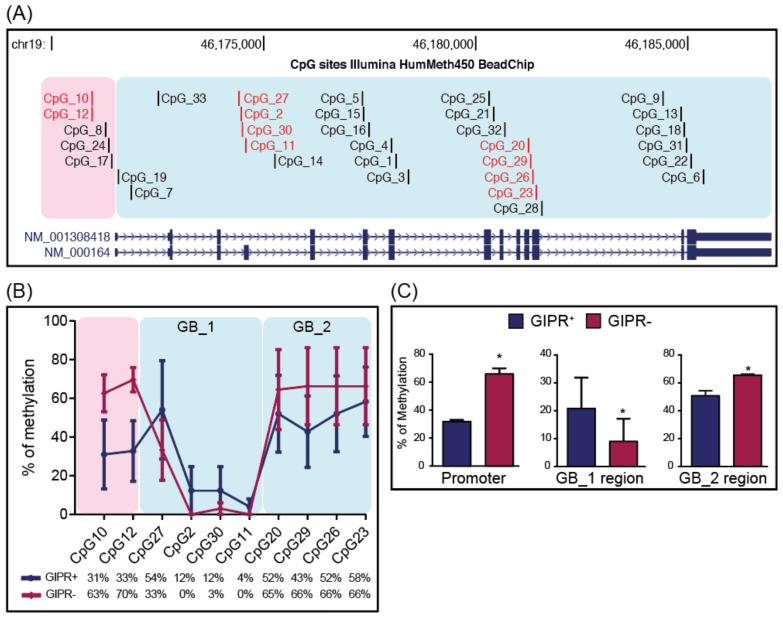
CpG sites in the GIPR locus of the HumMeth450 BeadChip were investigated by cloning-based BSP. (**A**) GIPR locus as annotated in the UCSC Genome Browser (GRCh37/hg 19 Assembly, genomic region chr19:46,169,919–46,187,927). Red CpG sites were investigated during this study. (**B**) The promoter and the gene body are reported as pink and pale blue shading boxes, respectively. The numbers below the graph refer to the percentage of methylation observed in each CpG analyzed in GIPR^+^ and GIPR^−^ somatotropinomas. (**C**) The percent methylation of the various CpG groups was compared between GIPR^+^ and GIPR^−^ GH-PAs. Values and error bars represent the mean of the percent methylation of CpGs within each cluster and the standard deviation of the data, respectively; significance: * *p* < 0.05.

To assess whether GIPR overexpression can be attributed to altered locus-specific methylation in the same way as some NETs [11], we analyzed CpGs within the *GIPR* locus on chromosome 19. Ten of the 27 CpG sites included in the HumMeth450 BeadChip, with a proven role in the modulation of *GIPR* expression [11] were selected and investigated by cloning-based Bisulfite Sequencing PCR (BSP) (Figure 2A). Based on their genomic location, the selected CpGs were clustered into 3 regions (Figure 2B). The first region partly overlaps the *GIPR* gene promoter, while GB_1 and GB_2 are located within the gene body.

We then compared the methylation levels of these clusters between the GIPR^+^ and GIPR^−^ groups (Figure 2B,C and Figure S1). We observed reduced methylation in the promoter region of the GIPR^+^ group when compared to the GIPR^−^ group (31.9% vs. 68.2%, *p* = 0.011) and opposite methylation patterns in the GB_1 and GB_2 regions. The GIPR^+^ group showed higher methylation levels in GB_1 (20.7% vs. 9.1%, *p* = 0.045) and lower methylation levels in GB_2 (51.2% vs. 65.8%, *p* = 0.021) if compared to GIPR^−^. Together, these findings suggest that GIPR expression may be influenced by epigenetic regulation in somatotropinomas.

### 2.2. Modulation of DNA Methylation Content in GH3 Cells Is Associated with Changes in Steady-State Levels of Gipr mRNA

To determine the extent to which promoter and gene body methylation contribute to the regulation of GIPR expression, we employed the mammosomatotrope cell line GH3. In this model, we altered the global DNA methylation content and evaluated whether these changes affected steady-state levels of *Gipr* and *Prl* mRNAs, using the latter as a positive control [23].

**Figure 3:**
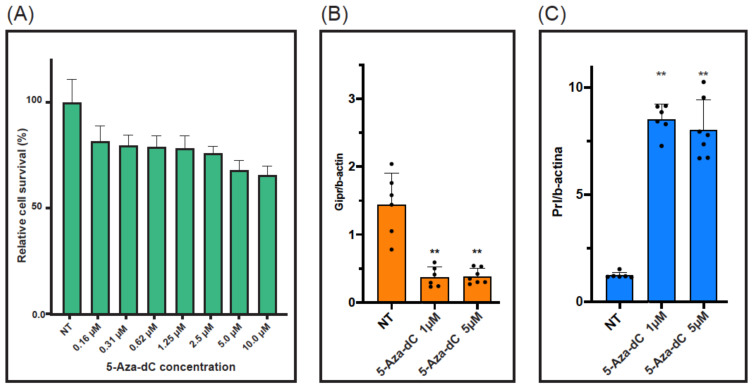
Effects of 5-Aza-dC treatment in GH3 cells. The effect of 5-Aza-dC on cell viability is reported in panel (**A**) as dose–response curves, 48 h after treatment. Cells were treated with 5-Aza-dC (1 μM and 5 μM) and the expression of *Gipr* (**B**) and *Prl* (**C**) were evaluated. Values and error bars represent the mean of three independent experiments in at least three replicates and the standard deviation of the data, respectively. ** *p* < 0.001 in treated vs. non-treated (NT) cells.

Initially, GH3 cells were treated with the DNA demethylating agent 5-aza-2′-deoxycytidine (5-Aza-dC), and gene expression was measured via RT-qPCR. We used two different concentrations (i.e., 1 μM and 5 μM), for which a limited cytotoxic effect was assessed (Figure 3A). 5-Aza-dC induced significant changes in the expression of the two genes without a dose–response effect (Figure 3B,C). The steady-state level of *Gipr* mRNA was significantly reduced four-fold compared to the control (for 1 µM, 1.44 ± 0.46 vs. 0.38 ± 0.15, *p* < 0.001). On the other hand, the *Prl* gene expression showed a seven-fold increase after drug administration (for 1 µM, 1.23 ± 0.15 vs. 8.51 ± 0.70, *p* < 0.001).

**Figure 4:**
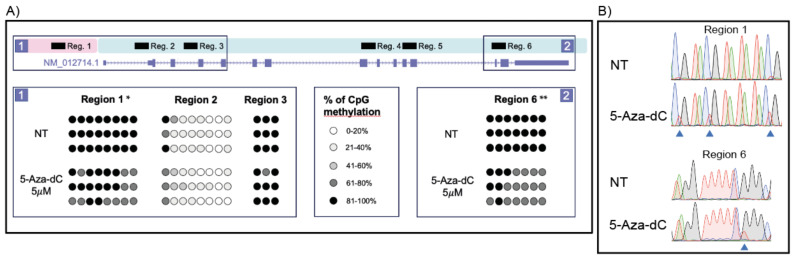
Direct BSP analysis of CpG sites in GH3 cells. (**A**) Schematic representation of the Gipr gene (UCSC Genome Browser RGSC 6.0/rn6, genomic region chr1:80,063,066–80,074,019), comprising the promoter (pink box), gene body (pale blue shading box), and the six regions investigated. In the bottom part of the panel, each row represents a single sample (i.e., NT and 5-Aza-dC) and each circle represents a single CpG in the PCR product. The percentage of CpG methylation is reported in different shades of gray. (**B**) Representative electropherograms of *Gipr* locus sequencing of bisulfite-converted DNA from treated (5-Aza-dC, 5 μM) and non-treated cells (NT). The triangles highlight the CpGs in which a methylation change has occurred. ** *p* < 0.001; * *p* < 0.05 in treated vs. NT cells.

Next, to determine the effects of the treatments on DNA methylation, six different regions of the *Gipr* locus, including a set of 54 CpGs, were investigated after 5-Aza-dC treatment by direct BSP (Figure 4). The selected CpGs were chosen based on their conservation and location in areas that partially overlap those of the human GIPR locus (see Section 4).

5-Aza-dC treatment did not induce any significant changes in DNA methylation at regions 2, 3, 4, and 5. It is worth noting that regions 4 and 5 were found to be completely unmethylated already under basal conditions. Significant changes were instead observed in regions 1 and 6, corresponding to the promoter and the 3′ section of the gene body. In particular, a significant reduction of 18% ± 2% and 26% ± 1% in the methylation rate was observed in regions 1 and 6, respectively (Figure 4).

## 3. Discussion

In recent years, aberrant expression of GIPR is being increasingly recognized in endocrine tumors, where it may also have relevant clinical implications [7]. Indeed, during meals, GIP/GIPR axis stimulation may result in the activation of molecular pathways involved in hormone synthesis and secretion [8,13,24]. Furthermore, this axis may be associated with specific phenotypic traits of diagnostic and/or prognostic relevance [7], which contributes to partly explaining the heterogeneity that characterizes each of these tumor types.

Recent evidence suggests that GIPR overexpression may take place as an early event in neoplastic transformation [8,25]. Therefore, gaining insight into the molecular mechanisms underlying this phenomenon can provide valuable information to elucidate the etiopathogenesis of these tumors.

In GH-PAs, the mechanism underlying GIPR overexpression and its role in neoplastic transformation has not been clarified yet. On the other hand, accumulating evidence demonstrates that GIPR modulates GH synthesis and secretion independently of the *gsp* oncogene [12,13,21] and that GIPR^+^ tumors belong to a specific transcriptional group, with a peculiar profile that includes *NR5A1*, *STAR*, and *CYP11A1* [14]. Moreover, it has been shown that GIPR^+^ tumors have a higher frequency of genomic rearrangements compared to their negative counterpart [12]. However, these alterations, as well as mutations in known (and predicted) regulatory regions, have been excluded as possible causes of GIPR overexpression [12,13]. A recent study reported an increase in global DNA methylation—particularly in gene bodies and outside the CpG islands—for GH-PAs. These tumors were also characterized by increased methylation of the *GIPR* gene body, suggesting that this may represent a driver event for the aberrant expression of *GIPR* in GH-PAs [12].

In the present study, considering only *GIPR* expression as a stratification criterion, we observed a profile of gene body hypermethylation in GIPR^+^ samples that is consistent with previous reports [11,12]. Furthermore, conversely from previous data obtained from GH-PAs [12] in these samples, we found reduced methylation of the promoter and of the 3′ of the gene body. Although the reasons for these differences are not immediately clear, the diverse analysis method employed (genome-wide methylation profiling in [13] vs. cloning-based BSP in the current work) and, more importantly, the distinct approach to sample stratification method (GIPR^+^/*gsp*^−^ vs. GIPR^−^/*gsp*^+^ adenomas in [13], while GIPR^+^ vs. GIPR^−^ in the current work) could account for such disparities. On the other side, while promoter hypomethylation has been traditionally associated with increased gene expression, growing evidence suggests that gene body hypermethylation may also play a significant role [26]. To determine to what extent promoter and gene body methylation may contribute to the regulation of GIPR expression, we used the rodent GH3 cell line, the only available option for this type of study. The 5-Aza-dC-based assay requires, indeed, actively proliferating cells, while primary cultures of somatotropinomas, which would have been a more adherent and reliable model, are not. Despite the limitations of using a rodent cell line that includes the partial correspondence of CpG sites at the GIPR locus between species, our findings indicate that gene body methylation may have a more significant impact than promoter methylation in this context. Indeed, we observed that 5-Aza-dC-induced hypomethylation specially on the gene body and a reduction in GIPR expression. On the basis of this evidence, it can be inferred that alterations in the methylation levels of the gene body are primarily responsible for the observed effect in cells of pituitary origin. If the effect were related to changes in GIPR promoter activity, we would have expected an increase in GIPR expression associated with this type of modification, which we did not instead. However, being an unselective treatment, which represents another limitation of this study, we cannot definitively affirm that the effect is locus-specific (see below). In any case, our data confirm that global modulation of methylation has an impact on GIPR expression, which has also been observed in other contexts. Treatment with benzo[a]pyrene, for example, can modulate the methylation of the entire GIPR locus and, at the same time, influence its expression [27,28].

On the other side, it is worth highlighting that the observed results may not be attributed solely to the demethylation effect of 5-Aza-dC, as DNA hypomethylating agents often have multiple biological targets and can be associated with an in-trans effect. Nucleoside analogs, for instance, have been shown to influence histone acetylation and/or DNA methylation. Hypomethylation mediated by 5-Aza-dC may indeed induce some genetic determinants of chromatin involved in gene expression regulation at genomic (translation repressor) or epigenomic levels (e.g., histone deacetylase SIRT6 [29], Dnmt3b [30]).

A further demonstration of the importance that epigenetic alterations may have as a cause of aberrant expression of GIPR has recently emerged in patients with FDCS [19,20]. In adrenal lesions of these patients, the loss of histone demethylase *KDM1A*, which acts in this context as a tumor suppressor gene, reduces the condensation of chromatin with subsequent overexpression of GIPR [19]. Recurrent germline mutations in *KDM1A* have never been reported in acromegalic patients [31,32,33,34]. This suggests that if alterations in this gene are actually involved in the differential expression of GIPR in GH-PAs, this is likely to occur with a different mechanism. GH-PAs show a higher frequency of chromosomal rearrangements [12,35]—including loss of a chromosome 1 region that harbors *KDM1A*—and this may suggest that somatic alterations affecting this gene could play a role in promoting GIPR overexpression in these tumors. On the other hand, in mouse ES cells, *KDM1A* is required for DNA methylation [36]. *KDM1A* interacts with DNA methyltransferases and thus possibly represents a key touchpoint between histone modification and DNA methylation in GH-PAs.

Assuming a more general perspective, elucidating the molecular mechanisms underlying diverse epigenetic alterations in somatotropinomas, including their interrelationships and their effects on chromatin instability and transcriptional profiles, is crucial for future research. The infrequent occurrence of genetic events, combined with data emerging from this and other recent studies, suggests that epigenetic alterations may play a fundamental role in the pathogenesis of somatotropinomas. Investigating, for instance, the presence of somatic alterations in *KDM1A* and their functional effects on GIPR expression, and more in general on the transcriptional profile of somatotropinomas, could be a promising target for short-term studies.

In conclusion, our study functionally supports previous evidence that epigenetic modulation can affect GIPR expression in GH-PAs. Nevertheless, it is worth mentioning that alterations in DNA methylation at the GIPR locus may only be a small part of a broader, more intricate regulatory process.

## 4. Materials and Methods

### 4.1. Patients and Tumoral Specimens

Portions of surgically removed specimens were immediately fixed in 10% buffered formalin and then embedded in paraffin. Standard sections stained with hematoxylin and eosin were used for diagnosis. Tumor specimens were examined for the presence of pituitary hormones using standard immunocytochemical analysis. The remainder of each somatotropinoma was immersed in RNAlater (Ambion, Milan, Italy), kept at 4 °C for 24 h, and then stored at −20 °C until use. Patients’ clinical features were reported elsewhere [13].

### 4.2. Cell Cultures and Modulation of Global DNA Methylation

The rat GH3 cell line (RRID: CVCL_0273, obtained from ECACC) was cultured in complete medium (CM) consisting of DMEM, 10% FCS, 2 mM l-glutamine, and penicillin/streptomycin, as described elsewhere [21]. All media and supplements were obtained from Gibco (Thermofisher, Milan, Italy).

DNA methylation content in GH3 cells was modulated by the DNA hypomethylating agent 5-aza-2′-deoxycytidine (5-Aza-dC, Merck, Milan, Italy).

To promote global hypomethylation, GH3 cells were seeded in 6-well plates in CM (1.5 × 10^5^ cells/well) and allowed to grow for 24 h, after which they were administered 5-Aza-dC (1 and 5 μM) or H_2_O as vehicle control. After 72 h, the cell medium was removed, cells PBS rinsed and both DNA and RNA were extracted as reported below. Working concentrations of 5-Aza-dC were established by testing its effects on the viability of GH3 cells, in 96-well plates, using the MTT proliferation assay (Merck, Milan, Italy), as described elsewhere [8].

### 4.3. Bisulfite Sequencing PCR (BSP)

The DNA methylation status of selected CpGs among nine GH-PAs and 5-Aza-dC-treated GH3 cells was assessed via cloning-based BSP and direct BSP, respectively, following established methodologies [37]. A total of 500 ng of genomic DNA extracted from nine frozen tumor specimens (QIAamp DNA Mini Kit, Qiagen, Milan, Italy) or GH3 cells (Quick-DNA/RNA Miniprep kit, Zymo Research) were treated with sodium bisulfite and recovered using the EZ DNA Methylation-Gold™ Kit (Zymo Research, Irvine, CA, USA) according to the manufacturer’s specifications.

The primers for the amplification of the selected CpGs were designed using Bisulfite Primer Seeker (https://www.zymoresearch.com/pages/bisulfite-primer-seeker, accessed on 9 November 2022), according to the following guidelines as reported elsewhere [37,38]: primer length ~30 bp, inclusion in the sequence of multiple C→T bases to ensure conversion specificity, amplicon length 300–400 bp, less than 3000 matches on the genome for primer specificity, exclusion of regions containing common SNPs.

For cloning-based BSP, we selected ten CpG of the Illumina HumMeth450 BeadChip (https://support.illumina.com/downloads/infinium_humanmethylation450_product_files.html, accessed on 20 April 2022). Since DNA is altered in bisulfite conversion, amplification and cloning bias can occur [39]. Consequently, to ensure proportional amplification and cloning of methylated and non-methylated DNA, for each locus, PCR and cloning conditions were established using a combination of fully methylated (FM) and non-methylated (NM) bisulfite-converted DNA (50% FM/NM) (Zymo Research). The loci of interest were amplified with ZymoTaq™ DNA Polymerase (Zymo Research). PCR products were cloned using the pGEM^®^-T Easy Vector System (Promega) or TOPO TA Cloning (Invitrogen) and transformed into DH5α (Invitrogen) bacteria. The plasmids were purified with the PureYield^TM^ Plasmid Miniprep System kit (Promega) according to the manufacturer’s specifications and sequenced using BigDye 1.1 Termination Chemistry on an ABI 3730XL (Applied Biosystems, Milan, Italy). Sequences obtained for each cloned allele showing a good-quality sequence (BS conversion > 90%) were scored for methylation status in each CpG. The proportion of methylated cytosine within the sequenced alleles was calculated by counting the number of alleles that showed methylation at the CpG sites of interest and dividing this by the total number of sequenced clones (n ≥ 8).

Within the *Gipr* locus, a set of 23 CpGs was chosen for direct BSP, which were conserved among human, rat, and at least one other species, such as dog, elephant, or chicken (sequences and alignments were retrieved from Human Genome Browser, https://genome.ucsc.edu/, accessed on 4 November 2022). These CpGs, along with 31 additional low-conserved CpGs, were included in 6 amplicons, amplified using ZymoTaq™ DNA polymerase (Zymo Research), and directly sequenced as previously described. Sequences obtained showing good-quality sequences (BS conversion > 90%) were scored for methylation status at each CpG. The proportion of methylated cytosine within sequenced alleles was calculated by comparing in the electropherograms, for each CpG, the relative heights of the C to T peak [37].

All primers and PCR conditions are available upon request.

### 4.4. RNA Extraction, Reverse Transcription, and Quantification

Total RNA from frozen tumor samples was extracted using TRIzol Reagent (Invitrogen, Milan, Italy) combined with the PureLink^®^ RNA Mini Kit (Ambion, Thermofisher). Instead, GH3 cells were processed using the Quick-DNA/RNA Miniprep kit (Zymo Research) according to the manufacturer’s instructions.

The integrity and yield of RNA were evaluated by an Agilent 2100 bioanalyzer (Agilent Technologies, Santa Clara, CA, USA) and a Nanodrop spectrophotometer (NanoDrop Technologies, Wilmington, DE, USA), respectively. Before reverse transcription, RNA samples were treated with the Turbo DNA-free kit (Ambion, Thermofisher) to remove DNA contamination. RNA was reverse-transcribed with M-MuLV Reverse Transcriptase RNase H- (Invitrogen) with random hexamers following the manufacturer’s instructions.

Absolute quantification of *GIPR* in GH-PAs and steady-state level of mRNA in GH3 cells were determined by digital droplet PCR (ddPCR) and RT-qPCR, respectively. Unless otherwise specified, all chemicals used in the droplet digital PCR and RT-qPCR assays were from Bio-Rad (Milan, Italy) and Applied Biosystems (Milan, Italy), respectively. ddPCR was performed as reported elsewhere [8]. Briefly, after dividing the cDNA mixture (i.e., ddPCR™ Super Mix for probes, target (*GIPR*, Hs00609210_m1) and reference (*HMBS*, Hs00609296_g1) amplification primer/probe mix, and 10 ng of cDNA template) into 20,000 droplets through a QX200™ Droplet Generator, samples were amplified (10 min at 95 °C, 40 cycles of 30 s at 94 °C, and 1 min at 60 °C, and a final step at 98 °C for 10 min). All cycling steps were performed with a ramp rate of 2 °C/s. Fluorescence was read on the QX200 Droplet Reader and analyzed by QuantaSoft™ Software v.1.7.4. For each sample, the data are reported in arbitrary units (i.e., the ratio of *GIPR*/*HMBS* mRNAs).

RT-qPCR experiments were performed following the MIQE guidelines. The GoTaq Probe qPCR Master Mix (Promega, Madison, WI, USA) and TaqMan Gene Expression Assays for *Gipr* (Rn00562325_m1) were used in an ABI PRISM 7900HT Sequence Detector. Prolactin gene (NM_012629; For 5′-ccgtgtggtcatgctttctc-3′; Rev 5′-ggaagaagtggggcagtcat-3′, working concentrations 300/50 nM) was instead assessed with the GoTaq qPCR Master Mix (Promega) on the same device. All samples were tested in duplicate on a MicroAmp 96-well reaction plate sealed with an optical adhesive film (Applied Biosystems) with 10 ng of cDNA in a 20 µL final reaction mixture. No-template controls were included in each run. The PCR conditions were 95 °C for 2 min, followed by 45 cycles at 95 °C for 15 s and 60 °C for 1 min. Data were analyzed with SDS rel.2.4 (Applied Biosystems), with an automatically set baseline and a fluorescence threshold adjusted for measuring quantification cycle (Cq) values. Validation experiments performed using the standard curve method with five serial dilutions of genomic DNA from control subjects showed matching amplification efficiencies (100% ± 10%) calculated according to E = 10^1/−slope^ − 1, for all assays.

As Β-actin levels remain unaffected by changes in DNA methylation, the amount of each target gene relative to this reference (Bact, Rn00667869_m1) was determined using the standard curve method.

### 4.5. Statistical Analysis

Statistical analyses were performed with the freely available R software (www.r-project.org, accessed on 10 January 2023) version 4.1.2. Two-tailed unpaired Student’s *t*-test with a 95% confidence interval was performed to compare the methylation status of the CpG sites in GIPR^+^ and GIPR^−^ GH-PAs. For cell culture studies, all experiments were performed at least twice, and results are presented as the mean ± SD of at least three determinations. The significance was determined by Mann–Whitney–Wilcoxon (MWW). The significance level was set at *p* < 0.05 for all tests.

## Data Availability

The data presented in this study are available within the article or upon reasonable request from the corresponding author.

## Supplementary Materials

The following supporting information can be downloaded at: https://www.mdpi.com/article/10.3390/ijms24119264/s1.
