# Supplementary material for: The Methylation Analysis of the Glucose-Dependent Insulinotropic Polypeptide Receptor (GIPR) Locus in GH-Secreting Pituitary Adenomas
Source: Int J Mol Sci. 2023 May 25;24(11):9264. doi: 10.3390/ijms24119264 (PMC10252571; doi:10.3390/ijms24119264)
Supplement: Supplementary file 1 [file ijms-24-09264-s001.zip › ijms-2380879-supplementary.pdf]

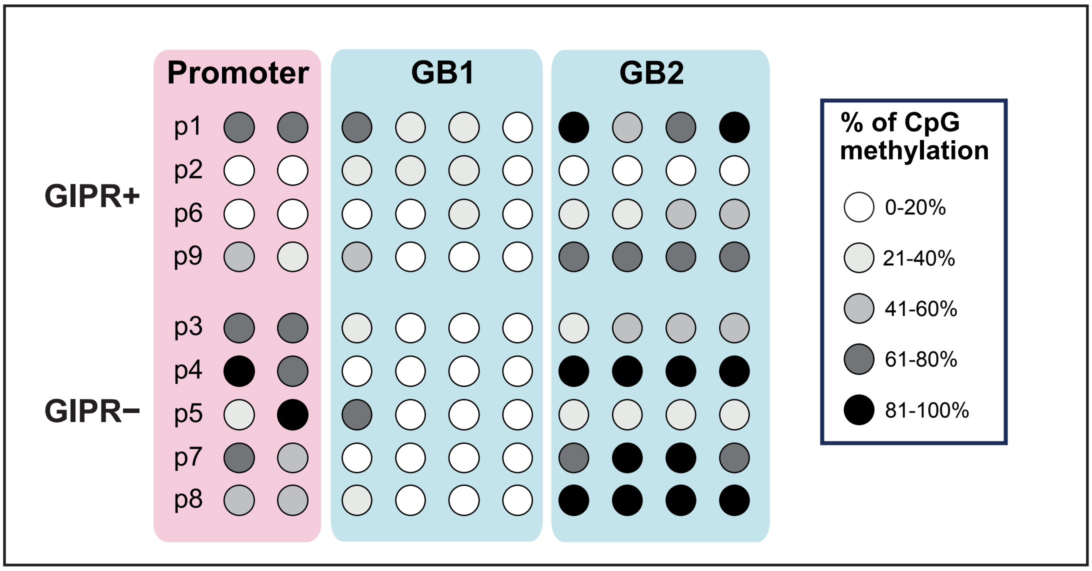

Supplementary Figure S1: cloning-based BSP analysis of CpG sites within GIPR locus in nine selected somatotropinomas. CpGs were located either in the promoter (pink box) or in two distinct regions of the gene body of GIPR (pale blue shading boxes). Each row represents a single sample, with GIPR+ samples shown in the upper part of the figure and GIPR- samples shown in the lower part. Each circle represents a single CpG analyzed. The percentage of CpG methylation is reported in different shades of gray and was calculated by dividing the number of alleles that showed methylation at each specific CpG site by the total number of sequenced clones (n≥8).
